# CD20+ B Cell Depletion in Systemic Autoimmune Diseases: Common Mechanism of Inhibition or Disease-Specific Effect on Humoral Immunity?

**DOI:** 10.1155/2014/973609

**Published:** 2014-03-27

**Authors:** Panagiotis Pateinakis, Athina Pyrpasopoulou

**Affiliations:** ^1^Department of Renal Medicine, Papageorgiou Hospital, 56403 Thessaloniki, Greece; ^2^2nd Prop Department of Internal Medicine, Hippokration General Hospital, Konstantinoupoleos 49, 54642 Thessaloniki, Greece

## Abstract

Autoimmunity remains a complex physiologic deviation, enabled and perpetuated by a variety of interplayers and pathways. Simplistic approaches, targeting either isolated end-effectors of more centrally placed interactors of these mechanisms, are continuously tried in an effort to comprehend and halt cascades with potential disabling and deleterious effects in the affected individuals. This review focuses on theoretical and clinically proved effects of rituximab-induced CD20+ B cell depletion on different systemic autoimmune diseases and extrapolates on pathogenetic mechanisms that may account for different interindividual or interdisease responses.

## 1. B Cell Maturation and Function

The B cell compartment is a vital constituent of the immune system and the key contributor to humoral immunity, a major branch of adaptive immunity. B cells belong to the group of white blood cells known as lymphocytes but can be distinguished from other subtypes of lymphocytes by the presence of the B-cell receptor on their surface. B-cell receptors are membrane-bound immunoglobulins and represent the key molecules involved in B cell activation. B cells develop from immature cells in the bone marrow, nurturing from stromal cells, which provide the appropriate environment for this process. B lymphocytes can be subdivided into different populations (B1, B2) that differ in development, surface marker expression, tissue localization, and function. B1 cells appear during fetal life and express surface IgM but little or no IgD. They arise in the bone marrow but renew their population by proliferation in the spleen and lymph nodes and in smaller quantities in the peritoneal and pleural cavities [1, 2]. B2 cells, also known as conventional or follicular (FO) B cells, are abundant in the spleen, lymph nodes, and peripheral blood and arise continuously from bone marrow precursors. They represent the major population of B lymphocytes, carry both IgM and IgD in their naive state, undergo negative selection to class antigens to prohibit autoimmunity, and mature through class switch.

Put in simple terms, the different B-cell lineage subsets throughout their maturation process include pro-B cells, pre-B cells, immature and transitional B cells, mature naive B cells, memory B cells, plasmablasts, and plasma cells. Plasmablasts are recently differentiated antibody-producing cells that are usually short-lived but can recirculate and settle in tissues such as the mucosa or the bone marrow, where they can differentiate into fully mature plasma cells [3, 4].

The leucocyte lineage derived subsets are hallmarked by specific clusters of differentiation markers (CD markers), cell surface molecules which serve as receptors or ligands in the pathways of cell signaling and in cell adhesion. The B cell-specific antigen CD20 is expressed during B cell development, starting at the pre-B cell level (but not found on stem cells or early pre-B cells), and further through B cell differentiation, only to become lost during terminal differentiation to plasma cells [5]. The CD system markers are commonly used in immunophenotyping, allowing cells to be defined based on what molecules can be identified on their surface. These markers are often used to associate specific cells with certain immune functions. One CD molecule, although uncommon, may be used to define cell populations; the combination of markers, however, has aided in the characterization of cell types with very specific definitions within the immune system.

## 2. The Role of B Cells in Autoimmunity

The immune mechanisms implicated in the development of autoimmune diseases have been categorized into two broad sets of diseases: one set in which the pathological process is driven mainly by T cells and the other in which the humoral B response mainly mediates the disorder by producing autoantibodies that are able to bind tissue self-antigens or by forming immune complexes [6].

B cell tolerance is important in preventing the development of antibody responses to protein antigens. Both central and peripheral mechanisms are implicated in B cell tolerance [7]. Immature B lymphocytes that recognize self-antigens with high affinity are deleted in the bone marrow; alternatively, mechanisms become activated to change their specificity by receptor editing. Intermediate binding affinity will permit B cells to survive and continue to the periphery where it may or may not promote autoimmunity, depending on interactions with other components of the immune system machinery [8, 9].

B cell contribution to autoimmunity is not confined to the production of autoantibodies. In animal models, whose B lymphocytes cannot secrete antibodies but can present antigens, autoimmune diseases (e.g., lupus) develop spontaneously; the ability of B cells to bind autoantigens through their B-cell receptor allows them to act as potent antigen presenting cells at very low protein concentrations [10].

Other functions of B cells, implicated in the pathogenesis of autoimmune diseases, include the production of cytokines and chemokines with immunosuppressive, polarizing, inflammatory, and tissue-organizing properties, actively modulating both humoral and cellular immune responses, and the neogenesis/formation of ectopic lymphoid tissue. Cytokines and chemokines produced by other cells, such as stromal, myeloid, or other cell types, promote survival and proliferation of pathogenic autoimmune B cells, thus perpetuating autoimmunity [11].

## 3. Rituximab: A Monoclonal Ab Targeting CD20 Positive B Cells

Rituximab, a mouse/human chimeric IgG1 mAb, was the first B-cell targeting therapeutic antibody used in clinical practice. It was originally developed for the treatment of chemotherapy resistant B-cell malignancies [12]. The clinical benefit that was observed in haematology patients with concomitant autoimmune diseases (rheumatoid arthritis) led initially to its clinical testing and its subsequent application in the treatment of these diseases.

Rituximab has a molecular weight of 145 kD and is composed of two heavy chains of 451 amino acids and two light chains of 213 amino acids. It targets the B-cell-restricted surface antigen CD20 with a binding affinity of approximately 8.0 nM [13] leading to rapid and profound B-cell depletion. Indeed, much knowledge gained about B cell depletion over the past decade has derived from preclinical and clinical data derived from the use of rituximab.

Apart from its direct impact on the specific elimination of CD20+ B-cells, rituximab appears to exhibit its immunomodulatory functions via nonspecific mechanisms, either through induction of complement-dependent cytotoxicity, as a nonspecific immunoglobulin administered intravenously, or through stimulation of the apoptotic pathway [14, 15].

Based on its broad potentials in the modulation of B-cell mediated immune mechanisms, rituximab was destined to become a candidate treatment in many immune-mediated diseases, with varying efficacy even among different manifestations of single clinical syndromes, thus provoking scientific interest about the complexity of the interactions among the components of the immune system within different clinical contexts.

## 4. Effect of Rituximab on B Cell Populations: Applications in Clinical Practice

To understand the potential effect, or lack of, B cell depletion therapy on B-cell centered auto-immune diseases, different B cell subtypes and their function/interaction with other components of the immune system need to become fully characterized. The effect of B cell depletion treatments can be imprinted through characterization of B cell subpopulations pre- and posttreatment and the net clinical effect assessed accordingly.

In broad terms, human B cells have been classified on the basis of 4 major surface markers (CD19, IgD, CD38, and CD27). CD19 is the earliest B specific protein expressed on B cells and becomes lost as the activated B cell matures into a plasma cell.

The IgD/CD27 classification builds on the notion of CD27 as a universal marker of human memory B cells to distinguish between memory cells (CD27+) and naive B cells (CD27−/IgD+). In turn, CD27+ memory cells can be divided into IgD+ (unswitched memory; usually together with IgM) and IgD− (switched memory; predominantly IgG+ or IgA+) [16]. The effect of rituximab on different CD20+ B cell subpopulations has been assessed in analogous clinical studies. Within the context of specific clinical syndromes, for example, rheumatoid arthritis, rituximab has been shown to be more efficient in subcategories of patients, regarding the specific disease, in seropositive patients [17]. To investigate the immunological imprint of this clinical observation, investigators compared the effects of rituximab on B cells from RF+ and RF− RA patients. In the CD27+ IgD–, memory subset, and CD27– IgD+, naive subset, rituximab significantly induced apoptosis in B cells from RF+ patients compared to untreated cells. In the CD27+ IgD+, double-positive B-cell subset, rituximab did not induce significant apoptosis either in the RF+ or in the RF− group.

Over the last few years several investigators have demonstrated the existence of important populations of CD27− memory cells. With the aid of multicolor flow cytometry additional heterogeneity within both CD27+ and CD27− memory cells could be demonstrated [18].

The clinical and immunological outcome of B cell depletion therapy will depend on the relative balance of protective and pathogenic B cell subsets established upon B cell repopulation. Even when the total B-cell count returns to normal, there appears to be a change in the phenotype, with the B cells present being relatively deficient in expression of CD27, that is, primarily naive, at least as late as 2 years after a single dose [19, 20]. Disease relapse in rheumatoid arthritis patients, on the other hand, was associated with the detection of blood memory B cells during repopulation.

## 5. The Effect of Rituximab on Antibody Production (Humoral Immunity)

Immunoglobulins are a central element in host defense. Moreover, many autoimmune diseases are characterized by the production of autoantibodies that are either directly responsible for cell or organ damage and/or diagnostic for the disease. The pathogenetic mechanisms of these diseases render them potentially responsive to B-cell targeted therapies.

Immunoglobulins are exclusively produced by B cells after antigen-mediated activation of their innate B-cell receptor. B cells are subsequently transformed to plasma cells and committed to immunoglobulin secretion.

B1 and marginal zone B cells can also secrete IgM. B1 cells have limited diversity of their B-cell receptor due to lack of the enzyme terminal deoxynucleotidyl transferase (TdT) and are potentially self-reactive. Antibody production by B1 cells is only associated with activation by very strong stimuli (such as bacterial proteins) and usually not by normal tissue proteins. However, especially in mice, where B1 cells represent a significant fraction of B cells, they have been implicated in the secretion of various autoantibodies, characteristic of autoimmune diseases.

The effect of rituximab on both pathological autoimmune B cell products in association with its clinical effect on the control of the relevant diseases, as well as on products of acquired B cell immunity, has been the subject of many clinical studies. Indeed, efficient B cell (CD19+) depletion has been found to correlate with reduction in the levels of certain autoantibodies (IgM rheumatoid factor) in seropositive rheumatoid arthritis, but not in the levels of ACPA autoantibodies in the same disease [21]. The assumption that this effect may be disease specific and antibody-type-associated is refuted by analogous studies in autoimmune diseases characterized by autoantibody production and autoantibody-mediated pathogenesis with contradicting effects on autoantibody levels in relation to response to treatment and disease activity. Control of disease activity in lupus, Sjogren's, and MPA patients could not be directly related to corresponding effect on autoantibody levels despite the drug's moderate to substantial effect on clinical improvement [22, 23].

Interestingly, immunization-related antibody responses, attributed to long-lived plasma cells even though transiently reduced during the treatment period, do not appear to be permanently affected.

Can this differential effect of rituximab on autoantibody titers and disease activity be thus solely attributed to the relative contribution of short- and long-lived plasma cells to the spectrum of each clinical situation, or could the B-cell related pathogenesis of every disease and the effect of rituximab extend beyond autoantibody production? [24]. In support of a more complex (autoantibody-independent) total effect of B cell depletion on immune-mediated pathways and their reflection on autoimmunity, administration of rituximab was additionally shown to affect T cell populations [25].

## 6. Rituximab in the Treatment of Systemic Autoimmune Diseases

### 6.1. RTX in Rheumatoid Arthritis

Rituximab was approved in 2008 in patients with rheumatoid arthritis. It is indicated for patients with moderate to severe disease that did not respond adequately to disease-modifying antirheumatic drugs, including the anti-tumor-necrosis-factor (TNF) biologics, and is rated as equally effective in comparison to a second TNF inhibitor [26]. The approved regimen includes 1000 mg of rituximab IV on days 0 and 14; retreatment is typically administered at 6 months, not sooner than 4 months, and adjusted to clinical response.

Seropositive rheumatoid arthritis was one of the first autoimmune diseases in which rituximab was clinically tried, based on the autoantibody-associated nature of the disease. The inclusion of seronegative patients in subsequent studies verified a less favorable outcome compared to seropositive patients. Clinical response was associated with efficient depletion of CD20+ B-cells and a reduction in IgM Rf but not ACPA titers. Moreover, relapse was invariably associated with B-cell repopulation. The response of seronegative rheumatoid arthritis patients, even though less impressive, is indicative of the multifaceted implication of B-cells in the pathogenesis of the disease (e.g., generation of immune complexes that act as decoys to attract monocyte/macrophages and thus reduce their inflammatory activity in certain autoantibody-mediated processes) [27, 28].

### 6.2. RTX in Systemic Vasculitis

Despite reports of successful treatment of refractory Takayasu arteritis, polyarteritis nodosa, and Kawasaki disease, rituximab appears to be, among vasculitic syndromes, most efficacious in the treatment of small vessel vasculitides [29]. CD20+ B cell depletion has been verified through well-controlled studies to efficiently control disease in both refractory and relapsing granulomatosis with polyangiitis [30, 31]. Clinical response to treatment, as well as disease activity per se, appeared to correlate, even though not invariably, with ANCA titers [32]. Inversely, inefficient peripheral blood B cell depletion and presumably peripheral tissue B cell depletion as well as residual ANCA titers appeared to associate with early disease relapses.

Interestingly, in a study showing encouraging results of rituximab treatment in patients with refractory HCV-related cryoglobulinemia, relapse was not associated with corresponding increases of the levels of cryoglobulins or to B cell repopulation per se, despite the reduction in disease activity and the decrease in cryoglobulin levels observed with efficient B cell depletion [33].

### 6.3. RTX in SLE

Based on the central role of autoantibodies and the multifaceted involvement of B cells in lupus pathogenesis, rituximab appeared as a potentially effective therapeutic agent in this setting.

Initial open, nonrandomized studies showed improvements in both clinical and laboratory features of disease following treatment with rituximab in refractory SLE [34]. Results, however, were not as encouraging in two large randomized, controlled SLE trials, which did not meet their endpoints in either renal or nonrenal lupus [35].

The different response of individual SLE patients to rituximab may reflect the heterogeneous pathogenesis of the disease among individual patients. In a study of rituximab administration in SLE patients with active disease [36], disease relapse was found to associate synergistically with B-cell numbers at repopulation and anti-dsDNA levels but not with the individual parameters per se. Relapse in these patients occurred with lower numbers of repopulating B cells than in the corresponding group. The rate of repopulation, however, in these patients did not differ in comparison to patients with lower levels of autoantibodies. The authors mention no correlation between B cell numbers and C3 levels.

SLE patients are characterized by disturbed B cell homeostasis, which translates into an increased population of plasmablasts and double negative (IgD− CD27−) B cells [37]. In the study by Lazarus et al. 2012, and in relation to anti-dsDNA antibody levels prior to the administration of rituximab, relapse was either characterized by increased percentage of IgD− CD27+ plasmablasts in patients with high levels of anti-ds DNA, or by increased percentage of IgD− CD27− (double negative) memory B cells at relapse in patients with low or normal levels of autoantibodies, respectively. These findings clearly indicate the need to identify biomarkers, which may reflect the different alternate disease mechanisms involved, and could identify which patients are more likely to respond to specific treatments [38, 39].

## 7. RTX: Different Mechanisms of Function for Different Seropositive Diseases?

The assumption that control of seropositivity may be the sole target of efficient treatment has well been disputed [40]. The removal of autoantibodies, identified and implicated in the pathogenesis of several autoimmune diseases, has proved beneficial, but not sufficient for the reduction of disease burden in most, if not all, cases. Of equal importance appears to be the efficient depletion of the pathogenic, autoimmune triggered/self-reacting CD20+ cells both in the peripheral blood, as well as localized in tissues and compartments, where they directly induce inflammation and initiate autoimmune cascades, the latter being a much less studied aspect of treatment effect. Switching at repopulation from autoimmunity to their naive state appears to be a vital contributor to the reprogramming of the immune system and the final effect of treatment.

The interplay and contribution, to various degrees, of other components of the immune system, such as the phagocytic compartment, in the presenting clinical syndromes may account for the variations of clinical response to rituximab within the different diseases, as well as for interindividual differences of response. Only once each individual patient can be biocharacterized, may the pathogenetic mechanisms of disease and response to treatment be deciphered.
